# Urine miR-21-5p as a potential biomarker for predicting effectiveness of tadalafil in benign prostatic hyperplasia

**DOI:** 10.4155/fsoa-2018-0012

**Published:** 2018-03-15

**Authors:** Tomoaki Tanaka, Akinori Minami, Kouichiro Tashiro, Naomasa Yoshida, Akira Tohda, Yasuo Yamakoshi, Ryoji Yasumoto, Shozo Sugita, Tatsuya Nakatani

**Affiliations:** 1 Department of Urology, Osaka City University Graduate School of Medicine, Osaka, Osaka, Japan; 2 Department of Urology, Meijibashi Hospital, Matsubara, Osaka, Japan; 3 Department of Urology, Yoshida Hospital, Hirakata, Osaka, Japan; 4 Department of Urology, Moriguchi Ikuno Memorial Hospital, Moriguchi, Osaka, Japan; 5 Department of Urology, Ishikiriseiki Hospital, Higashiosaka, Osaka, Japan; 6 Yasumoto Nephrology-Urology Clinic, Osaka, Osaka, Japan; 7 Department of Urology, Ohno Memorial Hospital, Osaka, Osaka, Japan

**Keywords:** biomarker, BPH, endothelial nitric oxide synthase, endothelium, LUTS, miR-21-5p, NO, PDE5, tadalafil, urine

## Abstract

**Aim:**

To investigate whether urine levels of miRNAs that regulate the function of endothelial cells are associated with effectiveness in benign prostatic hyperplasia (BPH) patients treated with a phosphodiesterase type 5 inhibitor, tadalafil.

**Patients & methods:**

We measured urine levels of three miRNAs (miR-21-5p, miR-126-5p & miR-155-5p) in 55 BPH patients before and after tadalafil administration to understand its effectiveness.

**Results:**

Baseline urine miR-21-5p level was an independent predictor of response to tadalafil in multivariate regression analysis (odds ratio: 0.28; 95% CI: 0.10–0.77; p = 0.014). Receiver operator curve analysis revealed that baseline urine miR-21-5p could serve as a predictor of response (area under curve: 0.85; 95% CI: 0.75–0.95; p < 0.001).

**Conclusion:**

Urine miR-21-5p could serve as a biomarker in predicting response of tadalafil for BPH.

## Lay abstract

Tadalafil, a phosphodiesterase type 5 inhibitor, has a high potential for treating male lower urinary tract symptoms; however, there are few reports regarding which portion of the population is appropriate for treatment with tadalafil. This study showed that the baseline urine levels of miR-21-5p, an endothelium-associated miRNA, strongly correlates with response to tadalafil and have the potential to be a predictive biomarker of whether treatment with tadalafil is suitable.

## Graphical abstract

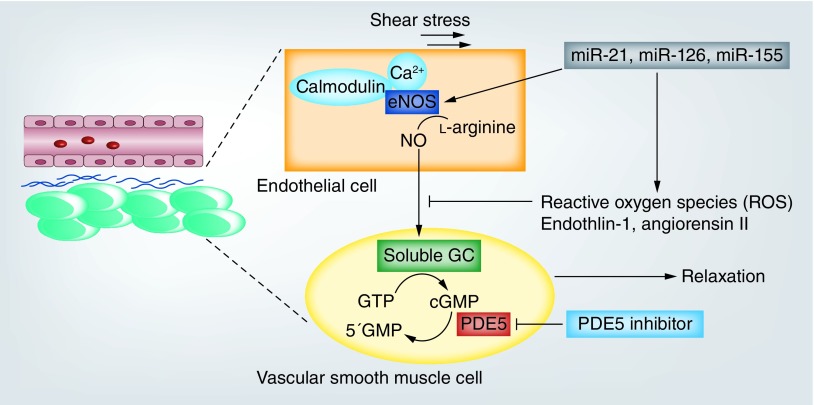

Male lower urinary tract symptoms (mLUTS) associated with benign prostatic hyperplasia (BPH) are a common complaint in senior adult men with a strong impact on quality of life (QoL), and substantial financial loss can result. The components of mLUTS include some symptoms, such as nocturia, increased urinary frequency, urgency, slow stream and sensation of incomplete emptying, in a voiding and bladder storage phase in urination. In general, some factors (e.g., insulin resistance, change in hormone balance, arterial sclerosis in pelvis, local inflammation) derived from aging and metabolic syndrome are believed to accelerate mLUTS with enlargement of prostate causing bladder outlet obstruction and bladder overactivity [1–3]. Although some medical treatments have been developed to target bothersome voiding or/and storage symptoms associated with mLUTS, one of the PDE5 inhibitors (PDE5-Is), tadalafil, is a brand-new drug for treatment of mLUTS/BPH and is recommended as a level A in worldwide clinical treatment guidelines owing to various excellent evidence [4,5]. PDE5-Is were reported to have some individual actions including the improvement of blood flow of pelvic viscera [6,7] and suppression of chronic prostatic inflammation [8], in addition to the release of bladder outlet obstruction [9] through the inactivation of PDE5/cyclic guanosine 3′ 5′-monophosphate (cGMP)/nitric oxide (NO) pathway. Recently, some reports revealed the crosstalk between mLUTS/BPH and endothelial dysfunction in microvessels [10,11]. MiRNAs are small, ncRNAs of 21–25 nucleotides that regulate gene expression via translational inhibition or activation of their target mRNAs. Various articles of research have indicated that some miRNAs play important roles in modulating the progress on endothelial dysfunction associated with atherosclerosis and ischemia [12]. In this study, we focused on three miRNAs (miR-21-5p [13,14], miR-126-5p [15] & miR-155-5p [16]) that possibly regulate the function of endothelial cells via the control of several genes. We investigated whether the expression pattern of these urine miRNAs is associated with the clinical effects in the mLUTS/BPH patients treated with tadalafil.

## Patients & methods

### Study population & urine collection

This clinical study protocol was approved by the institutional review board of Osaka City University. A total of 70 patients with mLUTS caused by BPH were enrolled in this study between 2014 and 2016. The ethics committees of the participating hospitals approved this study, and informed consent was obtained from each patient. Finally, a total of 55 patients, excluding the patients who discontinued the administration, were examined. The patients received the administration of a PDE5 inhibitor, tadalafil, 5 mg/day for 12 weeks and their spot urine was used for measurement of the expression of miRNAs before and at 4 weeks after the treatment. Each spot urine was collected in the urine collection and preservation tube (Norgen Bioteck Co., Ontario, Canada) and stored at room temperature before isolation of miRNAs.

### Evaluation of mLUTS in subjective & objective findings & definition of clinical response

Subjective urinary symptom score (International Prostate Symptom Score [I-PSS] and Overactive Bladder Symptoms Score [OABSS]), QoL and Nocturia-QoL index were estimated before and at 4, 8 and 12 weeks after administration of tadalafil. Objective urinary parameters (maximum urinary flow rate [Q_max_] and average urinary flow rate [Q_ave_]) in the uroflowmetry were measured before and at 12 weeks after the treatment. The clinically meaningful improvement (CMI) was defined as ≥25% baseline-to-endpoint total IPSS improvement; or ≥2.5 ml/s baseline-to-endpoint Q_max_ improvement.

### RNA isolation & reverse transcription & real-time polymerase chain reaction

*Caenorhabditis elegans* miR-39 (cel-miR-39) was used as external reference for assaying the miRNAs in urine [17]. Cel-miR-39 of 20 pmol/l was added as the spin-in control after the denaturing solution of 500 μl was added. Total RNA including miRNAs component was isolated and purified using the urine MicroRNA Purification Kit (Norgen Bioteck Co.) in accordance with the manufacturer's protocol. The reverse transcription was performed with 10 ng of total RNA and TaqMan MicroRNA Reverse Transcription Kit (Applied Biosystems, CA, USA). The target miRNA was quantified using TaqMan Universal Master Mix II (no UNG) and each TaqMan assay; has-miR-21-5p (ID: 000397), has-miR-126-5p (ID: 000451) and has-miR-155-5p (ID: 002623), according to the manufacturer's protocol (Applied Biosystems). Each reaction was carried out in a total volume of 20 μl containing 1.3 μl reverse transcription products, 1 μl 20× TaqMan assay primer, 10 μl 2× TaqMan Universal Master Mix II (no UNG) and nuclease-free water to adjust the volume. The PCR reaction was performed as follows: 95°C for 10 min, followed by 50 cycles of 95°C for 15 s and 60°C for 30 s. The comparative cycle threshold method (ΔCt) was exploited to calculate the relative expression level of miRNA. Mean Ct values and deviations between the duplicates were calculated for all samples. ΔCt = Ct (target miRNA) - Ct (Cel-miR-39) and relative miRNA expression were determined using the formula 2-ΔCt. In addition, each miRNA expression was set as the ratio against each urine creatinine level to avoid bias of urine concentration. The final values of miRNA levels were in logarithm scale.

### Statistical analysis

Statistical analysis was performed by the Ekuseru-Toukei 2015 software (Social Survey Research Information Co., Ltd, Tokyo, Japan). The nonparametric Mann–Whitney U test and χ^2^ test were used to analyze differences in the clinical characteristics and miRNAs abundances in two groups. The influence of explanatory variables on CMI was analyzed by means of the logistic regression analysis. Receiver operating characteristics curve was applied to analyze the prognostic value of urine miR-21-5p. Youden Index (sensitivity and 1-specificity) was used to identify the optimal cut-off threshold value. p-values <0.05 were considered statistically significant.

## Results

### Basic characteristics of BPH patients

Table 1 shows the clinical characteristics of responders (n = 36) and nonresponders (n = 19) based on the definition of CMI related with tadalafil treatment. There are no significant differences between two groups in age, BMI, prostate volume, total I-PSS, I-PSS voiding and storage subscore, QoL, Nocturia-QoL, OABSS, the severity grade of BPH and the rate of additional use on α1 adrenoceptor blocker.

**Table 1.** **Clinical characteristics and parameters of the patients.**

**Variable**	**Responder (n = 36)**	**Nonresponder (n = 19)**	**p-value**
Age (years)	68.5 ± 1.6	72.7 ± 1.6	0.069

BMI (kg/m^2^)	24.1 ± 0.6	24.0 ± 0.6	0.4

PV (ml)	30.0 ± 4.3	33.3 ± 5.9	0.43

***I-PSS***			

Total score	18.4 ± 1.5	16.3 ± 1.6	0.32

Storage subscore	8.2 ± 0.7	7.1 ± 0.7	0.34

Voiding subscore	10.2 ± 1.0	9.2 ± 1.3	0.48

QoL	4.7 ± 0.2	4.5 ± 0.2	0.41

OABSS	6.1 ± 0.7	5.2 ± 0.6	0.48

N-QoL index	15.3 ± 2.2	15.4 ± 1.9	0.82

***BPH severity ***			

Mild	5	3	0.76

Moderate	16	10	

Severe	15	6	

***Additional use of tadalafil on α1 adrenoceptor blocker***			

No	14	8	0.82

Yes	22	11	

Data were expressed as mean ± standard deviation.

BPH: Benign prostatic hyperplasia; I-PSS: International prostate symptom score; N-QoL index: Nocturia-quality of life index; OABSS: Overactive Bladder Symptoms Score; PV: Prostate volume; QoL: Quality of life.

### Comparison of responders & nonresponders regarding overactive bladder symptoms

**Figure 1.**
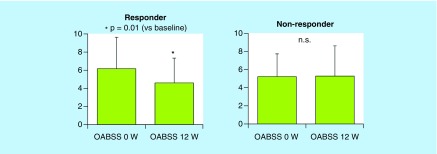
**In the clinically meaningful improvement responder group, the values of Overactive Bladder Symptoms Score in post-treatment 12 weeks were significantly lower than those in pretreatment 0 week.** In the clinically meaningful improvement nonresponder group, there was no significant difference between pre- and post-treatment 12 weeks Overactive Bladder Symptoms Score values. n.s.: Not significant; OABSS: Overactive Bladder Symptoms Score.

As shown in Figure 1, the OABSS values of responders were significantly reduced by treatment with tadalafil, but those of nonresponders were not significantly changed. The responders satisfied with CMI criteria also showed significant improvement in the scale of overactive bladder.

### Comparison of responders & nonresponders in the baseline levels of urine miRNAs

**Figure 2.**
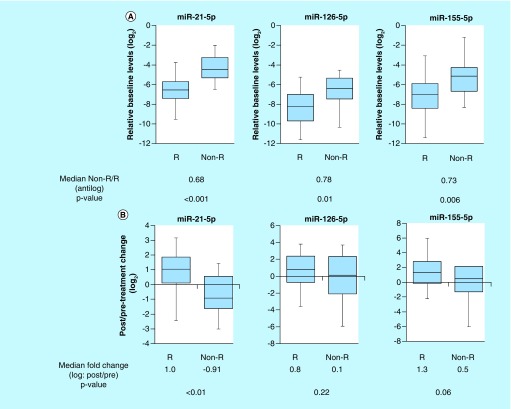
**Comparison of responders and nonresponders in urine levels of miR-21-5p, miR-126-5p and miR-155-5p.** **(A)** The baseline urine levels of miR-21-5p, miR-126-5p and miR-155-5p in responders were significantly lower compared with the baseline urine levels of them in nonresponders, respectively (p < 0.001, p = 0.01 and p = 0.006). **(B)** The change between the pre- and post-treatment 4 weeks in urine miR-21-5p levels of responders was significantly high compared with that of nonresponders (p < 0.01). The change between the pre- and post-treatment 4 weeks in either urine miR-126-5p or miR-155-5p levels of responders showed no significant difference from that of nonresponders, respectively. Non-R: Non-responder; R: Responder.

The results showed that the baseline urine levels of miR-21-5p, miR-126-5p and miR-155-5p in responders were significantly lower compared with the baseline urine levels of them in nonresponders, respectively (p < 0.001, p = 0.01 and p = 0.006) (Figure 2A).

### Comparison of responders & nonresponders in the changes between the baseline & post-treatment levels of urine miRNAs

The change of urine miR-21-5p expression between the baseline and post treatment 4 weeks in responders was significantly high compared with that in nonresponders (p < 0.01) (Figure 2B). After tadalafil treatment, responders tended to have an increase in post-treatment levels of urine miR-21-5p. There are no significant differences between responders and nonresponders in alterations of either urine miR-126-5p or miR-155-5p due to tadalafil administration, respectively (p = 0.22, 0.06) (Figure 2B).

### Univariate & multivariate repression analyses of parameters associated with urine miRNAs expression

First, there is a no significant impact factor in several clinical parameters (i.e., age, BMI, prostate volume, I-PSS, OABSS, QOL, etc.) in univariate analysis. As shown in Table 2, the results showed that each baseline levels in urine miR-21-5p, miR-126-5p and miR-155-5p and the ratio of post-/pretreatment urine levels of miR-21-5p have a significant impact on CMI of treatment with tadalafil in univariate models. Furthermore, the multivariate analysis confirmed the baseline levels in urine miR-21-5p as an independent predictor (OR: 0.28; 95% CI: 0.10–0.77; p = 0.014).

**Table 2.** **Possible predictors in response to tadalafil treatment – logistic univariate and multivariate regression analyses.**

**Variables**	**Univariate models**		**Multivariate models**	

	***OR (95% CI)***	***p-value***	***OR (95% CI)***	***p-value***
Baseline urine miR-21-5p	0.4 (0.23–0.7)	0.001	0.28 (0.10–0.77)	0.014

Baseline urine miR-126-5p	0.65 (0.47–0.89)	0.0083	1.00 (0.58–1.8)	0.97

Baseline urine miR-155-5p	0.68 (0.5–0.92)	0.012	1.57 (0.81–3.1)	0.18

Δ urine miR-21-5p	4.3 (1.3–14.4)	0.018	1.36 (0.77–2.4)	0.28

Δ: Change between baseline and post-treatment levels of miR-21-5p (increased vs decreased/unchanged); OR: Odds ratio.

### Predictive value of urine miRNAs in mLUTS/BPH patients treated with tadalafil

**Figure 3.**
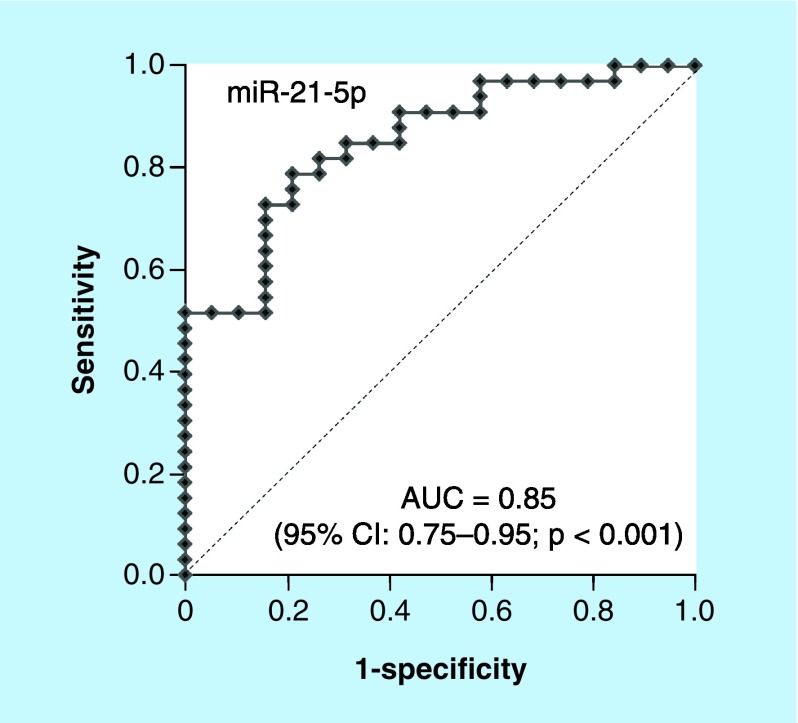
**The baseline urine miR-21-5p produced AUC of 0.85 (95% CI: 0.75–0.95; p < 0.001) and the optimal cut-off value was -5.7 with sensitivity and specificity of 78.8 and 78.9%, respectively.** AUC: Area under curve.

We performed receiver operating characteristics analysis to evaluate the predictive power of the baseline urine miRNAs levels for treatment response of tadalafil against mLUTS/BPH patients. The baseline urine miR-21-5p yielded AUC of 0.85 (95% CI: 0.75–0.95; p < 0.001) and the optimal cut-off value was -5.7 with sensitivity and specificity of 78.8 and 78.9%, respectively (Figure 3). In addition, the baseline urine miR-126-5p and miR-155-5p exhibited AUC of 0.73 (95% CI: 0.58–0.88; sensitivity = 72.7%, specificity = 68.4%, p = 0.003) and 0.73 (95% CI: 0.59–0.87; sensitivity = 84.8%, specificity = 57.9%, p = 0.002), respectively (Supplementary Figure 1).

## Discussion

The PDE5-Is have been reported to exert strong pharmaceutical effects for smooth muscle relaxant in bladder neck and prostatic urethra [9,18,19], vasodilatation response in lower urinary tract (LUT) [7,20], inhibition of bladder afferent nerve activity [21] and reduction in prostatic inflammation related with LUTS [8]. Although the functional role of PDE5 remains controversial, a main stream is NO/cGMP signaling pathway in the crosstalk between efferent nerve terminal and LUT smooth muscle cell, or endothelial and vessel smooth muscle cell [22]. Previous reports revealed that expression of PDE5 is robust in endothelial and smooth muscle cells of LUT vessels, and tadalafil plays a critical role in vasodilatation of vesicular-differential artery via inhibition of cGMP degradation by blocking of PDE5 activity [6,23]. PDE5-Is initially have been used on-demand in erectile dysfunction (ED) for the purpose of vasodilatation of penile cavernous arteries. Administration of tadalafil for ED patients was also reported to increase percentage flow-mediated dilatation as an indicator of endothelial function and induce circulating endothelial precursor cells [24,25]. In addition, a recent study reported that response of endothelium to tadalafil treatment significantly correlated to improvement of mLUTS [26].

While there is a large amount of scientific evidence for clinical response of PDE5-Is in mLUTS and ED, few reports investigating which subgroup of patients with mLUTS would represent preferable effectiveness of PDE5-Is have been found until now. If an appropriate biomarker for predicting the clinical response of PDE5-Is could be put into practical use, it would contribute to a development of personalized medical care for mLUTS/BPH.

To our knowledge, this study is the first report to explore whether urine levels of miRNAs associated with regulation of endothelial functions could predict the response to the PDE5-I tadalafil in mLUTS/BPH. Our study demonstrated that the pretreatment baseline levels of three urine miRNAs, and the change between the pre- and post-treatment level in urine miR-21-5p could differentiate responders from nonresponders to tadalafil. Finally, we provided evidence that the baseline urine levels of miR-21-5p exerted an independent predictive factor in logistic multivariate repression analysis. It has been reported that the expression of miR-21 is induced by blood shear stress in endothelium, and miR-21 increases the production of NO through upregulation of endothelial NO synthase and suppresses apoptosis of endothelium via inhibition of phosphate and tensin homolog deleted from chromosome 10 [13,14]. In a past study, it was demonstrated that reduction of endothelial-expressed miRNAs may be attributed to uptake into atherosclerotic lesions in patients with cardiovascular disease [27,28]. The population of patients that have endothelial dysfunction in LUT microvessels concomitantly with low levels of circulating endothelial miRNAs may be a good candidate for treating with tadalafil. On the contrary, Vlachopoulos *et al*. reported that mLUTS/BPH patients with cardiovascular disease risk, who were administrated >1 antihypertensive drug, have significantly lower IPSS improvement compared with men taking ≤1 drug [29]. Furthermore, they demonstrated that use of diuretics resulted in significantly lower IPSS improvement compared with men taking other antihypertensives or no drugs. The patients with severe atherosclerosis, who need some kinds of antihypertensive drugs, seem to be inappropriate for treatment with tadalafil because of irreversible endothelial dysfunction. Moreover, upregulation of miR-21 has been shown to lead to MAPK-dependent reactive oxygen species production and reduction of NO bioavailability in endothelial precursor cells [30]. Interestingly, antagonism of miR-21 was reported to improve dysfunction of angiogenic progenitor cells [31].

Although this study indicated that the baseline levels of urine miR21-5p below the cutoff -5.7 with sensitivity and specificity of 78.8 and 78.9%, respectively, may be a useful predictor for tadalafil treatment in mLUTS/BPH, there are some limitations of this study including no examination was performed with negative control miRNAs that do not target endothelium; numbers of examined BPH patients; and no measurements for clinical endothelial function values (e.g., percentage flow-mediated dilatation, reactive hyperemia index) or the blood flow velocity surrounding bladder in researched patients. Based on the current data, we will prospectively investigate whether urine miR-21-5p levels correlate with the values in endothelial function and vesical blood flow in BPH patients treated with tadalafil.

## Conclusion

In summary, we confirmed that urine expression patterns of miRNAs associated with endothelial function possibly correlate with effectiveness of tadalafil treatment in mLUTS/BPH patients. Particularly, the baseline urine levels of miR-21-5p could be a promising biomarker predictive for its response.

## Future perspective

All evidence from this study showed that urine levels of three endothelium-associated miRNAs (miR-21-5p, miR-126-5p, miR-155-5p) have a potential for correlation with the treatment response to tadalafil in mLUTS/BPH patients. In particular, the baseline urine level of miR-21-5p exerted an excellent prediction for effectiveness of tadalafil in those patients. The next step is to determine whether urine levels of these miRNAs also associate with endothelial function values and vesical blood circulation in a large number of patients. Finally, the determination of appropriate cut-off values of urine levels in endothelial miRNAs will lead to a tailor-made medication of tadalafil for mLUTS/BPH patients.

**Summary points**

- Each baseline urine level of miR-21-5p, miR-126-5p and miR-155-5p in tadalafil responders is significantly lower than those in tadalafil nonresponders, respectively.
- Change between baseline and post-treatment urine level of miR-21-5p in tadalafil responders is significantly higher than that in tadalafil nonresponders.
- Baseline urine levels of miR-21-5p are an independent factor predictive for tadalafil response.
- Baseline urine miR-21-5P yields AUC of 0.85 (95% CI: 0.75–0.95; p < 0.001) and the optimal cut-off value is -5.7 with sensitivity and specificity of 78.8 and 78.9%, respectively.
