# Supplementary figures and images for: Urine miR-21-5p as a potential biomarker for predicting effectiveness of tadalafil in benign prostatic hyperplasia
Source: Future Sci OA. 2018 Mar 15;4(6):FSO304. doi: 10.4155/fsoa-2018-0012 (PMC6060390; doi:10.4155/fsoa-2018-0012)

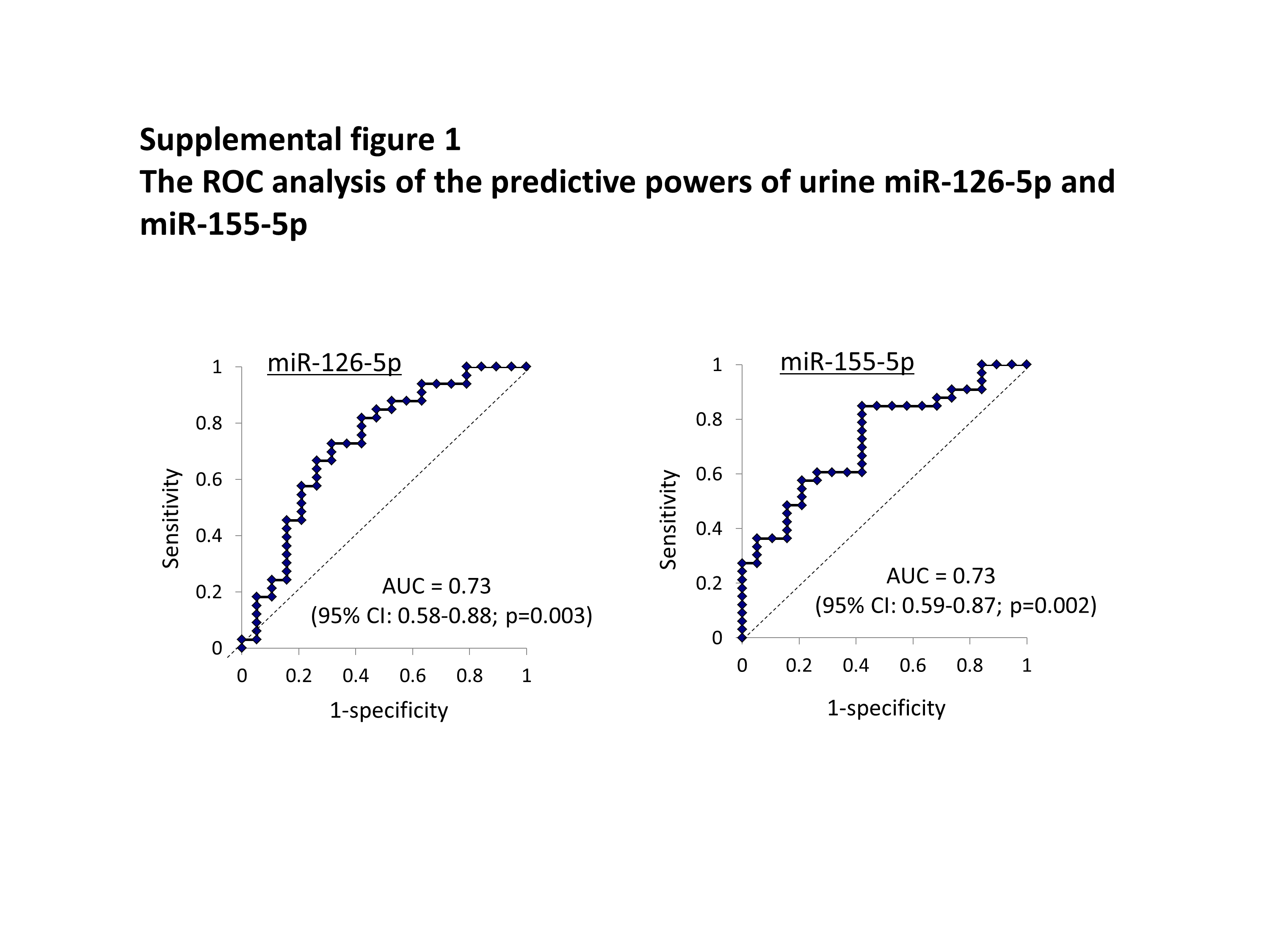

Supplement: Supplementary file 1 [file fsoa-04-304-s1.tif]
